# Expression of Separate Proteins in the Same Plant Leaves and Cells Using Two Independent Virus-Based Gene Vectors

**DOI:** 10.3389/fpls.2017.01808

**Published:** 2017-11-07

**Authors:** Maria R. Mendoza, Alexandria N. Payne, Sean Castillo, Megan Crocker, Brian D. Shaw, Herman B. Scholthof

**Affiliations:** Department of Plant Pathology and Microbiology, Texas A&M AgriLife Research, Texas A&M University, College Station, TX, United States

**Keywords:** plant, virus, gene vector, TMV, TBSV

## Abstract

Plant viral vectors enable the expression of proteins at high levels in a relatively short time. For many purposes (e.g., cell biological interaction studies) it may be desirable to express more than one protein in a single cell but that is often not feasible when using a single virus vector. Such a co-expression strategy requires the simultaneous delivery by two compatible and non-competitive viruses that can co-exist to each express a separate protein. Here, we report on the use of two agro-launchable coat-protein gene substitution GFP-expressing virus vector systems based on *Tomato bushy stunt virus* (TBSV) referred to as TG, and *Tobacco mosaic virus* (TMV) annotated as TRBO-G. TG expressed GFP in *Nicotiana benthamiana*, tomato, lettuce and cowpea, whereas expression from TRBO-G was detected only in the first two species. Upon co-infiltration of the two vectors co-expression was monitored by: molecular detection of the two slightly differently sized GFPs, suppressor-complementation assays, and using TG in combination with TRBO-RFP. All the results revealed that in *N. benthamiana* and tomato the TBSV and TMV vectors accumulated and expressed proteins in the same plants, the same leaves, and in the same cells. Therefore, co-expression by these two vectors provides a platform for fast and high level expression of proteins to study their cell biology or other properties.

## Introduction

Expression of foreign proteins in plants is normally achieved via transformation with *Agrobacterium tumefaciens* or by biolistics, to generate stable transgenic plants within several months by incorporating foreign DNA into the plant chromosome. But, an alternative technique involves the use of virus systems to express proteins in plants, which has the advantage of fast expression and high yields within a few days after infection, and consequently relatively high quantities can be isolated from infected plants (Scholthof et al., 2002). Several plant viruses have been designed to serve as vectors for expression of foreign proteins and the most promising to date are DNA-based viruses (Hefferon, 2014) or single-stranded (ss) positive-sense RNA viruses, as demonstrated for instance for one or more members in the genera *Tobamovirus*, *Potexvirus*, *Tobravirus*, and *Comovirus* (Porta and Lomonossoff, 2002; Scholthof et al., 2002; Burch-Smith et al., 2004; Gleba et al., 2004, 2007; Lindbo, 2007; Liu and Kearney, 2010a,b).

One technical disadvantage associated with virus-mediated protein expression is that due to size constraints with regards to the insertion of foreign material, most viruses only support the expression of one protein (Gleba et al., 2004), while many bioactive complexes are often composed of oligomers of different proteins. Also, using the same viral vector construct as backbone for different proteins in co-infections often can lead to one construct interfering with or outcompeting the other (Gleba et al., 2007). This phenomenon is also referred to as superinfection exclusion (Folimonova et al., 2010), which probably contributes at the molecular level to the control method of cross protection (Hull, 2002). Overcoming these limitations requires the use of at least two non-competitive plant viral vectors to achieve expression of more than one protein. For example, such a strategy was reported by co-expressing two polypeptides using vectors based on *Tobacco mosaic virus* (TMV) and *Potato virus X* (PVX) (Giritch et al., 2006) that is used to produce pharmaceutical vaccine.

The present study addressed the question whether *Tomato bushy stunt virus* (TBSV) and TMV virus vectors can express foreign proteins at high levels in the same cells of different plant species. These two viruses were chosen because of their high accumulation and concomitant gene expression (Scholthof et al., 1999; Lindbo, 2007); the absence of a biological transmission vector that could otherwise raise biosafety concerns (Hull, 2002; Scholthof, 2004; Yamamura and Scholthof, 2005); they can be very effectively introduced upon agroinfiltration (Lindbo, 2007; Shamekova et al., 2014); and they both express effective suppressors of RNA silencing, each with a different mode of action (Omarov and Scholthof, 2012) that could potentially lead to extra gene expression stimulation when combined.

**FIGURE 1:**
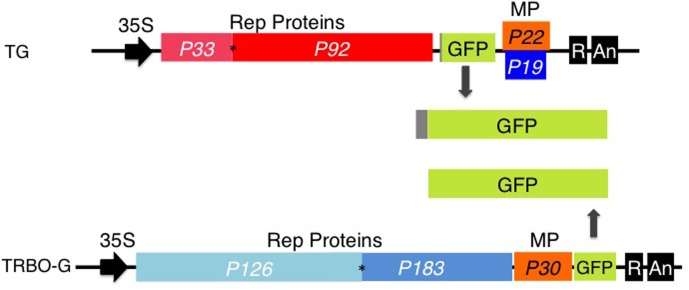
TBSV-GFP (TG) and TRBO-GFP (TRBO-G) viral vectors. Only the relevant part of the insert in the binary vectors is illustrated. *In vivo* transcription of the viral (+) sense RNA genomes is driven by the upstream CaMV 35S (35S) promoter; the dark box labeled ‘R’ at the 3′ end represents a ribozyme, ‘An’ refers to the poly (A) signal. The numerical “P” notations indicate the size in kDa of the proteins encoded by the viral genes. Rep Proteins indicate replication-associated proteins; MP, cell-to-cell movement protein; P19, suppressor of RNA silencing. GFP expressed from TG contains 17 extra amino acids that correspond to the remnant N-terminal end of the capsid protein. The asterisks indicate the presence of an amber read-through stop-codon. Further details on TG and TRBO-G were published previously (Lindbo, 2007; Shamekova et al., 2014).

Over the years we have explored the gene vector utility of TBSV, a (+)sense ssRNA virus with an icosahedral structure (Yamamura and Scholthof, 2005). Previous results have shown that agro-launchable coat protein gene substitution GFP-expressing TBSV constructs (TG; **Figure 1**) successfully infect agroinfiltrated leaves of *Nicotiana benthamiana* and cowpea (Shamekova et al., 2014). A promising vector based on the (+)sense ssRNA genome of the rod-shaped TMV (Lindbo, 2007), is represented by TRBO-G (**Figure 1**), which has the coat protein replaced with GFP to yield high levels of protein expression in agroinfiltrated leaves (Lindbo, 2007) (**Figure 1**).

At the onset of this study it was somewhat of a surprise that we failed to find any reports in the literature determining whether TBSV and TMV can co-infect plants, even though these viruses have been studied for a more or less a century (Hull, 2002; Scholthof, 2004). Whether such mixed infections with TBSV and TMV occur in the same plant is an interesting biological question in itself but also in the context of using virus vectors and therefore we examined this property using TRBO-G and TG. The results show that both vectors TG and TRBO-G can infect *N. benthamiana* and tomato whereas, unlike TRBO-G, TG is also able to express GFP in lettuce and cowpea. Furthermore, in *N. benthamiana* and tomato TRBO-G (or TRBO-RFP) and TG can co-infect at the whole plant, leaf, and cellular levels, enabling the expression two separate proteins in the same cells of infected plants.

## Materials and Methods

To prepare infiltration cultures of the viral vector constructs, *Agrobacterium* GV3101 containing a viral construct was grown at 28°C overnight in LB media containing 50 mg/L kanamycin. Then, a subculture was prepared to grow bacteria cultures in LB media with 50 mg/L kanamycin, 20 μM acetocyringone, and 10 mM MES. The day of infiltration bacteria were collected by centrifugation at 4000 rpm for 20 min, the supernatant was discarded and bacteria pellets were resuspended at 0.5 600 OD in infiltration media (10 mM MgCl_2_, 10 mM MES pH 5.6, and 200 μM acetosyringone). After cultures were suspended in infiltration media, they were incubated for 4–6 h in the dark at room temperature. Leaves of plants were infiltrated at the abaxial side using 1 ml syringe.

*Nicotiana benthamiana* was infiltrated at 3–4 weeks, and based on preliminary tests, it was determined that cowpea required 1-week old plants, Grand Rapids lettuce 2-week old plants, and tomato 3-week old plants. Plants were grown in a growth chamber with 60% humidity, 22°C for 16 h under light, 20°C for 8 h under dark. The treatments for these experiments were: Mock (infiltration buffer only), TRV-00 (which contains an empty TRV vector), TG and TRBO-G constructs. After infiltration, plants were monitored for 3–5 days and 50 mg of plant leaf samples were collected for protein expression analysis. GFP imaging and western blot analyses were performed as previously described (Odokonyero et al., 2015).

To determine co-expression, TRBO-RFP was constructed. For this, TRBO-G was digested with *Pac*I and *Not*I to remove the GFP gene and this was substituted with the RFP sequence amplified with the same restriction enzyme sites at the termini, using standard PCR and cloning techniques. Similarly, a TRBO construct devoid of GFP was used that instead expressed a single-guide RNA for the N-gene; this construct served as the TRBOdG (not expressing GFP) construct used in complementation assays. Agroinfiltration with these constructs was performed at 0.3 600 OD together with TG. Infiltrated plants were visualized under the fluorescent microscope for GFP and RFP expression. The versions of GFP are as previously published (Lindbo, 2007; Shamekova et al., 2014) and the RFP was removed from pSITE-4NB (Chakrabarty et al., 2007) and recloned.

Microscopy was performed on Olympus BX51 microscope (Olympus America, Melville, NY, United States) and images were captured with Hamamatsu Orca-ER cooled CCD camera (Hamamatsu, Japan). For GFP visualization an Olympus U-MNIBA2 filter cube with excitation wavelengths from 470 to 480 nm, emission wavelengths from 510 to 550 nm, and a dichroic mirror at 505 nm. For RFP visualization an Olympus U-MNIBA2 filter cube was used with excitation wavelengths from 350 to 550 nm, emission wavelengths from 590 to 630, and a dichroic mirror at 570 nm. Images were acquired using Slidebook Version 5, which controlled a Prior shutter (Prior Scientific, Rockland, MA, United States). During the multiple microscopic imaging experiments it was verified that green fluorescence was only associated with TG infection and red fluorescence with TRBO-RFP infection; in other words no green fluorescence was associated with excitation and/or emission of RFP, and no red fluorescence could be attributed to GFP.

## Results

### Comparison between TBSV and TMV Vectors in Different Hosts

**FIGURE 2:**
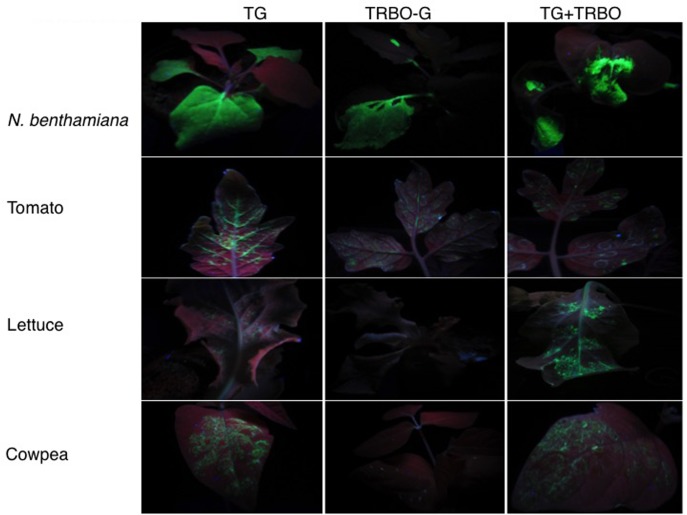
GFP expression upon *Agrobacterium* mediated infiltration of viral vectors in 4 week-old *Nicotiana benthamiana*, 3 week-old tomato, 2 week-old lettuce, and 1 week-old cowpea. The constructs used for infiltration are indicated on top. GFP expression was visualized with UV illumination at 3 days post-infiltration (dpi).

*Nicotiana benthamiana* is a common experimental plant to study virus-mediated expression of proteins (Goodin et al., 2008). However, other plant species can be desirable, for instance to study the biochemistry and cell biology of specific proteins in different host backgrounds. Previous work in the laboratory had pointed toward tomato, lettuce, and cowpea as good expression platforms for TBSV (Seaberg et al., 2012). However in those experiments the virus was inoculated as RNA, which has much lower inoculation efficiency than the agroinfiltration technique used in subsequent studies (Shamekova et al., 2014). Therefore, to compare expression between TG and TRBO-G we agroinfiltrated *N. benthamiana*, tomato, lettuce, and cowpea with these constructs. The results showed that in lettuce and cowpea the GFP expression was readily observed in leaves infiltrated with TG, but no expression was observed in these plants for TRBO-G (**Figure 2**). On the other hand *N. benthamiana* and tomato plants supported GFP expression from both TG and TRBO-G, whether inoculated separately or together (**Figure 2**). Initial time-course studies (Supplementary Figures S1–S4) showed that, based on GFP intensity, the GFP expression was relatively high for both vectors in *N. benthamiana* while lower in tomato (Supplementary Figures S2, S3) and no obvious additive effect was noted upon co-infection (Supplementary Figure S4).

### Co-expression by the TMV and TBSV Vectors in the Same Tissue

**FIGURE 3:**
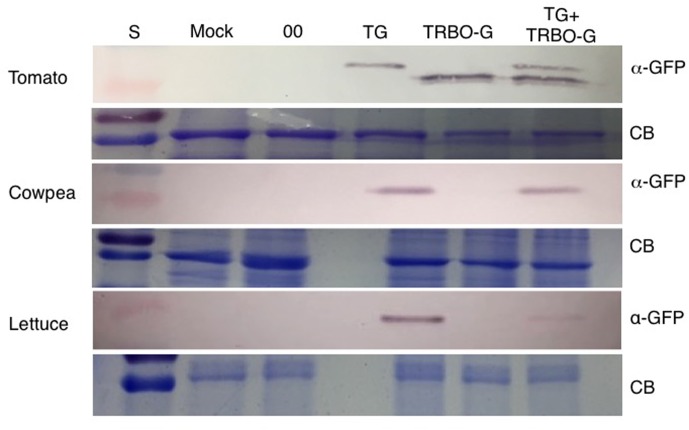
Western blot for detection of GFP in tomato, cowpea and lettuce. TG samples were collected at 3 dpi while Mock, 00 (control *Agrobacterium* expressing an empty TRV vector), TRBO-G and TG+TRBO-G were collected at 7 dpi. Primary GFP antibodies were used (α-GFP), and Coomassie Brilliant Blue (CB) staining was performed to provide loading comparisons. The red colored size marker (S) on the left of western blots is 25 kDa, while the size of the intense CB band of a host protein is about 55 kDa as inferred from the size marker on the left.

The GFP images of **Figure 2** and Supplementary Figures S1–S4 did not yet reveal whether both vectors were co-infecting and/or co-expressing, nor did it rule out that TRBO-G accumulated at low levels in cowpea and lettuce. Therefore, western blot analysis was performed for molecular detection and relative quantification of GFP expression from each vector. As depicted in **Figure 1** the molecular size of GFP expressed from TG was predicted to be somewhat higher than that expressed by TRBO-G because of its fusion with the remnant 17 N-terminal TBSV coat protein amino acids (**Figure 1**). This size differential provided a convenient tool to measure if upon co-infection both vectors indeed expressed GFP, as can be seen in tomato (**Figure 3**-top). In agreement with **Figure 2**, western blot analyses of agroinfiltrated cowpea and lettuce showed that GFP only accumulated in leaves infected with TG, for TRBO-G no GFP was detected (**Figure 3**).

**FIGURE 4:**
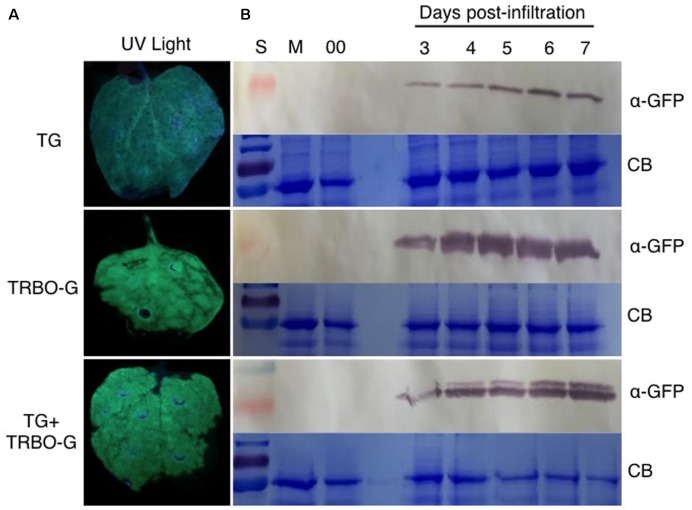
GFP analysis in *N. benthamiana* based on green fluorescence and western blot assays. **(A)** GFP imaging at 3 dpi in *N. benthamiana* infiltrated with TG and TRBO-G, TG+TRBO-G. **(B)** GFP protein analysis in *N. benthamiana* infiltrated as for **(A)**. GFP expression for TG, TRBO-G, and TG+TRBO-G was monitored each day from 3 to 7 days post-infiltration (dpi) with Mock (M) and 00 (control *Agrobacterium* expressing an empty TRV vector) collected at 7 dpi. In each case the upper panels are western blots for GFP detection, while the lower panels show the corresponding Coomassie Brilliant Blue (CB) staining of the gels for loading comparison. The red colored size marker (S) on the left of western blots is 25 kDa, while the size of the intense CB band of a host protein is about 55 kDa as inferred from the size marker on the left.

**FIGURE 5:**
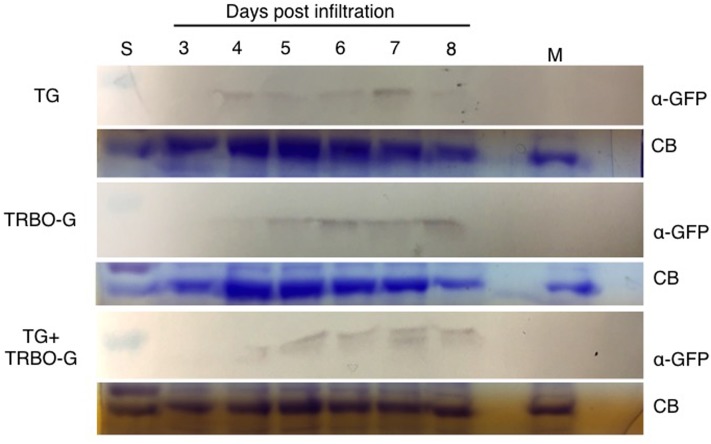
Time-course for GFP detection in tomato infected with vectors as indicated. Samples were taken from 3 to 8 dpi. Mock (M) was taken at 8 dpi. The red colored size marker (S) on the left of western blots is 25 kDa, while the size of the intense CB band of a host protein is about 55 kDa as inferred from the size marker on the left.

An infection time-course followed by western analyses with extracts from infected *N. benthamiana* (**Figure 4**) and tomato leaves (**Figure 5**) verified that GFP expressed from the TG vector has a slightly larger molecular mass compared to GFP from TRBO-G. Furthermore, the findings confirm the results of **Figure 2** that *N. benthamiana* and tomato are both hosts for TG and TRBO-G. Importantly, both GFP versions were detected when a co-infiltration was performed in *N. benthamiana* (**Figure 4**) and tomato and (**Figures 3**, **5**), providing evidence that each vector expressed GFP in co-agroinfiltrated leaves. Collectively, the results lead to the conclusion that both vectors co-exist, co-accumulate, and co-express proteins in the same infected tissue in *N. benthamiana* and tomato. Furthermore, in *N. benthamiana* TRBO-G gave higher levels of expression than TG (**Figure 4**), while this difference was less apparent in tomato (**Figure 5**).

The time-course analyses further showed that in *N. benthamiana* the TG vector expressed detectable amounts of GFP starting at day 3, and the protein intensity increases up to 7 days (**Figure 4**). After this time, infiltrated *N. benthamiana* leaves started to wilt and were no longer conducive for protein assays because of the necrotic effects of P19. In the case of TRBO-G, GFP was observed at 3 days post-infiltration, and reaching the highest level at 4 dpi, which was maintained until the end of the time course study (**Figure 4**). In tomato GFP accumulation became visible for both vectors at day 4 and expression was maintained until day 8 (**Figure 5**). The levels in tomato were lower than in *N. benthamiana* (**Figures 4**, **5**), in accordance with the difference in GFP intensities (Supplementary Figures S1–S4).

### Co-expression by the TMV and TBSV Vectors in the Same Cells

**FIGURE 6:**
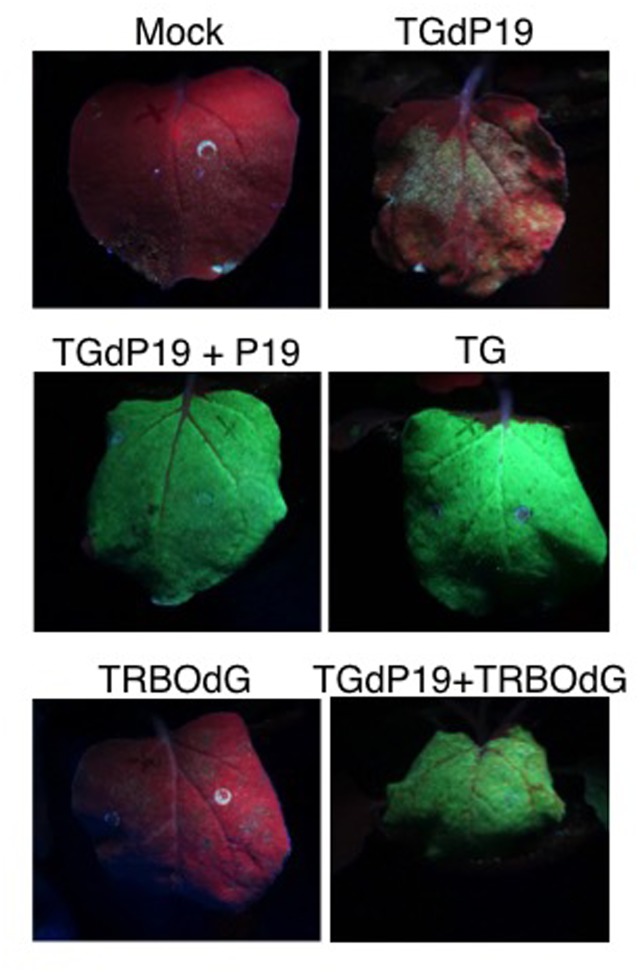
Co-infiltration of TGdP19 (TG not expressing P19) with TRBOdG (TRBO not expressing GFP) on *N. benthamiana*. Images were taken at 6 dpi. Top panels show infiltrated leaves for Mock and TGdP19; middle panels TGdP19+P19 and TG; and lower panels TRBOdG and TGdP19+TRBOdG.

The above experiments showed that TG and TRBO-G were co-expressing GFP in the same leaves. However, these analyses could not address whether both vectors were present and expressing in the same cells. Initial supportive data for the co-existence at the cellular level were acquired by suppressor-complementation assays using an equivalent of TG that lacks the ability to express the P19 suppressor (TGdP19) and is thus silenced for GFP expression (Shamekova et al., 2014). The complementation studies revealed that TGdP19 was not only rescued by co-infiltration with a construct expressing P19, but also by co-infection with a TRBO construct not expressing GFP (TRBOdG) (**Figure 6**). Apparently the suppressor activity associated with the replicase of TMV (Ding et al., 2004) complemented TGdP19 at the cellular level.

**FIGURE 7:**
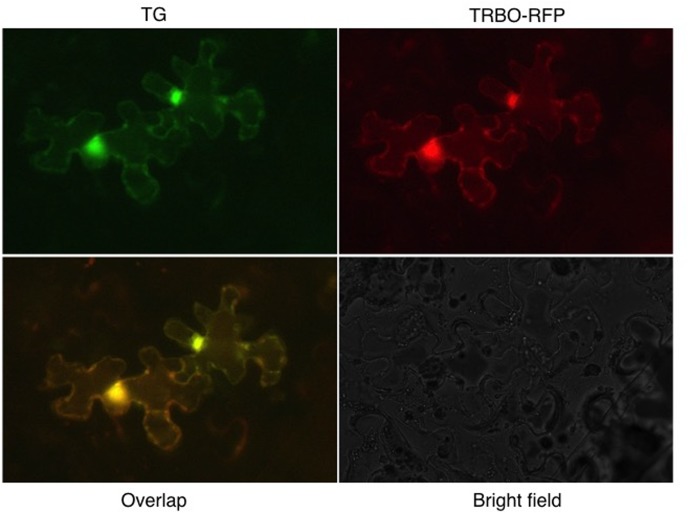
TG and TRBO-RFP co-agroinfiltrated on 4-week old *N. benthamiana*. Plant epidermal cells were observed 10 days after infiltration. Fluorescence occurs throughout the cytoplasm and the bright spots probably reflect accumulation of fluorescent components in the cytoplasm surrounding the nucleus.

To enable a direct cell biological approach, we aimed to have one of the two vectors express RFP instead of GFP, followed by-co-infiltrations to determine whether co-infection by the two TBSV and TMV vectors could be visualized. For this purpose, the GFP insert in TRBO-G was replaced with the RFP gene to yield TRBO-RFP. This was infiltrated in tomato leaves (Supplementary Figure S5) individually or in combination with TG and the results, even though of low resolution, supported the notion that co-infections occurred. More detailed and higher contrast fluorescence microscopy was conducted with *N. benthamiana* and that unequivocally confirmed that TG-mediated GFP expression and TRBO mediated RFP expression were readily apparent in the same cells as visualized for select epidermal cells (**Figure 7**). This was inferred from the orange fluorescence, as would be expected by overlaying green and red fluorescence. Therefore, the collective results of the present study showed that TG and TRBO vectors co-express in the same plant, the same leaves, and in the same cells.

## Discussion

In the present study, the performance of TMV- and TBSV-based gene expression vectors (TG and TRBO-G, respectively) was monitored either individually or in combination, in different plant species. The findings showed that TG and TRBO-G are capable of infecting and expressing GFP in *N. benthamiana* and tomato, but only the TG vector was able to infect and express in cowpea and lettuce. Visual fluorescence inspections and more quantitative immunoblot comparisons indicated that compared to TG, TRBO-G expression generally occurs at higher levels in *N. benthamiana* while this difference for the two vectors is less evident in tomato. For each vector, GFP accumulation in tomato was inferior to that observed for *N. benthamiana*. Therefore, depending on the host, TRBO-G performance is either superior or equal to that of TG, but the added utility of the TG system is the property that it can be used for a wider range of plant species (**Figures 2**, **3**) (Yamamura and Scholthof, 2005; Seaberg et al., 2012). Even though direct comparisons are lacking, the expression from virus vectors is generally higher than that observed for non-replicating 35S promoter driven constructs because of the cell-to-cell spread. It also deserves mentioning that neither TG nor TRBO moves systemically over long distance and thus expression is limited to the inoculated leaves.

Using various tests, such as imaging assays performed under UV-light, western blot analyses, suppressor-complementation assays, and cell biology using fluorescent microscopy, all provided evidence that TG and TRBO-G co-express their respective foreign proteins in the same leaves, tissues, and cells in *N. benthamiana* and tomato. Only a few other reports present results that two different backbone virus vectors can co-exist in the same host (Gleba et al., 2004, 2007) but no such information was available for TBSV and TMV. However, our results reveal that a TMV-based vector can be combined with a TBSV-based vector to co-express two different proteins at high levels. Therefore, during the time-span of our experiments (3–8 days) one construct did not outcompete another construct through silencing or any other competitive mechanism but rather they co-existed. Part of this outcome may be related to the fact that both vectors express a suppressor (replicase for TMV and P19 for TBSV) each with a different mode of action (Omarov and Scholthof, 2012).

Another interesting feature is that even though both TBSV and TMV have been extensively studied the past century (Hull, 2002), thus far we have not been able to find information on co-infections in nature or under experimental conditions. In the present study the TMV and TBSV coat protein gene replacement vectors were forced together by agroinfiltration and this demonstrated that at the molecular level there is no hindrance for both viruses to accumulate in the same plants, leaves, and cells. It is possible that co-infections also readily occur in nature but that this simply has not been reported. On the other hand, our observations do lead to a question whether in nature perhaps some unknown property, for instance coat protein expression, is affecting their ability to co-persist?

## Conclusion

We have provided novel evidence that TBSV- and TMV-derived gene expression vectors can co-exist at the whole leaf, tissue, and cellular levels in plants. This opens up possibilities to co-express peptides and proteins that form bioactive oligomers. Another important aspect is that the high levels of expression provide a convenient and rapidly applicable means for biochemical characterization and detailed high-resolution cell biological studies on proteins that normally are expressed at much lower levels.

## Author Contributions

MM designed and conducted experiments and wrote the manuscript. AP, SC, and MC assisted with experiments and provided data. BS assisted with microscopy and image capturing. HS advised during all stages of the project and co-wrote and edited the manuscript.

## Conflict of Interest Statement

The authors declare that the research was conducted in the absence of any commercial or financial relationships that could be construed as a potential conflict of interest.
