# Supplementary figures and images for: Expression of Separate Proteins in the Same Plant Leaves and Cells Using Two Independent Virus-Based Gene Vectors
Source: Front Plant Sci. 2017 Nov 7;8:1808. doi: 10.3389/fpls.2017.01808 (PMC5681929; doi:10.3389/fpls.2017.01808)

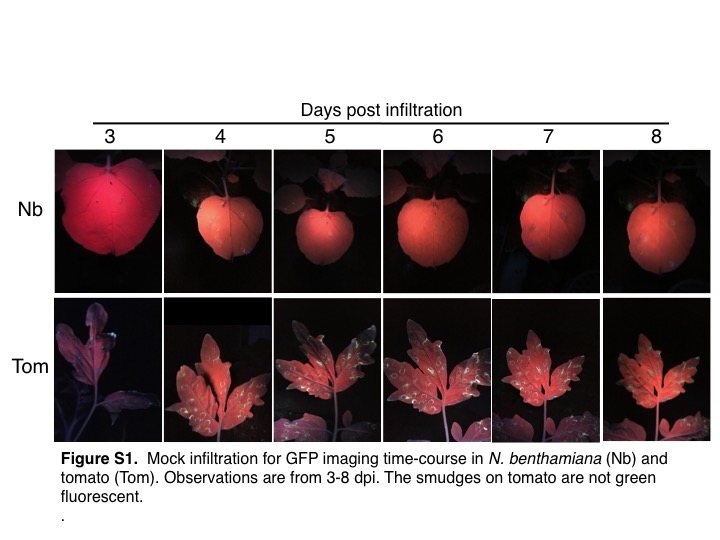

Supplement: Supplementary file 1 [file Image_1.JPEG]

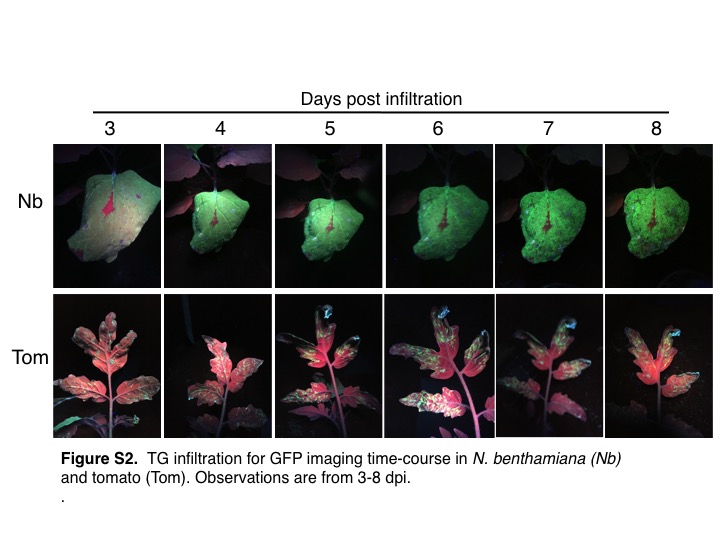

Supplement: Supplementary file 2 [file Image_2.JPEG]

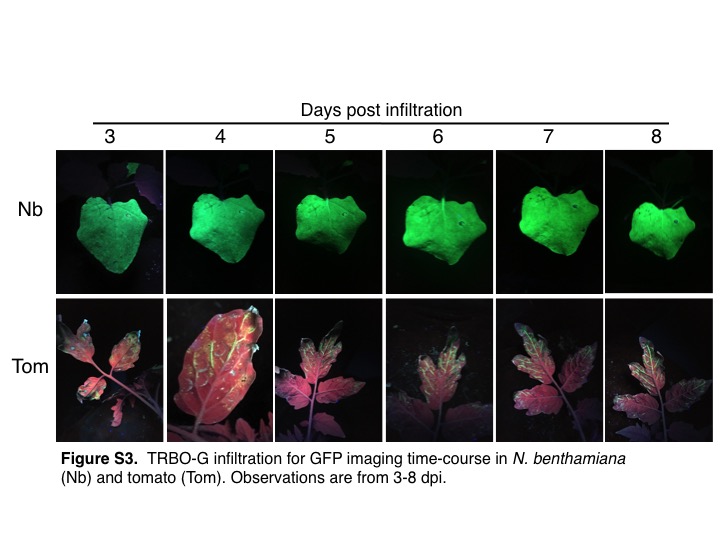

Supplement: Supplementary file 3 [file Image_3.JPEG]

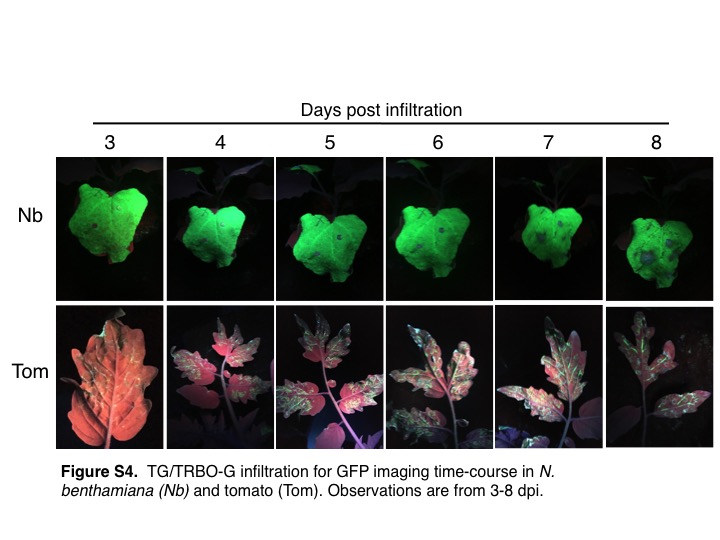

Supplement: Supplementary file 4 [file Image_4.JPEG]

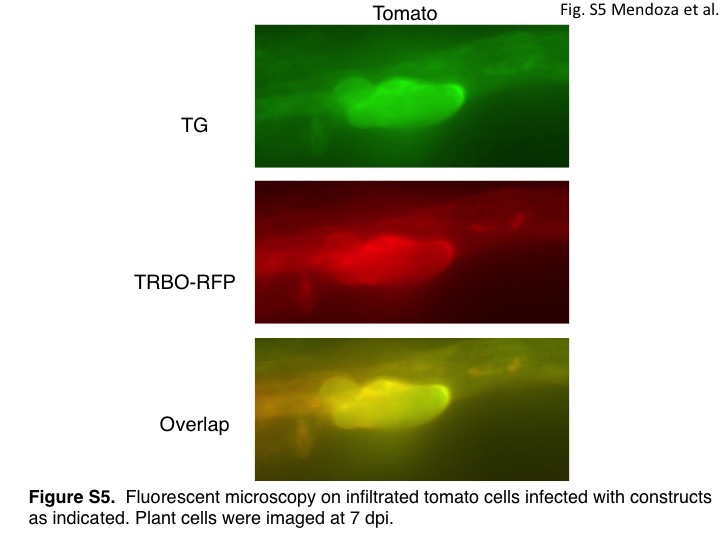

Supplement: Supplementary file 5 [file Image_5.JPEG]
